# BDNF genetic variants modulate the impact of childhood trauma on symptom dimensions in first-episode schizophrenia

**DOI:** 10.3389/fpsyt.2026.1790184

**Published:** 2026-03-05

**Authors:** Junjiao Ping, Yong Wu, Jiali Luo, Ying Zhang, Tingyun Jiang, Yonghui Dang

**Affiliations:** 1 College of Medicine and Forensics, Xi’an Jiaotong University Health Science Center, Xi’an, Shanxi, China; 2 Department of Psychiatry, The Third People’s Hospital of Zhongshan, Zhongshan, Guangdong, China

**Keywords:** *BDNF*, childhood trauma, clinical symptoms, gene polymorphism, schizophrenia

## Abstract

**Background:**

Gene–environment interactions play a critical role in shaping phenotypic heterogeneity in complex psychiatric disorders. Brain-derived neurotrophic factor (BDNF) is a key genetic regulator of stress-sensitive neuroplasticity. Yet, how *BDNF* polymorphisms are associated with the effect and impact of childhood trauma on clinical phenotypes remains incompletely understood.

**Methods:**

We conducted a case–control study including 93 patients with first-episode schizophrenia (SZ) and 64 healthy controls. Childhood trauma exposure was assessed using the Childhood Trauma Questionnaire (CTQ), and symptom dimensions were evaluated with the Positive and Negative Syndrome Scale (PANSS). Three *BDNF* single-nucleotide polymorphisms (rs6265, rs2030324, and rs11030101) were genotyped. Generalized linear models were applied to examine gene–environment interaction effects while adjusting for demographic and clinical covariates.

**Results:**

Patients with SZ exhibited significantly higher CTQ scores across all trauma subtypes compared with controls (all *P* < 0.05). Childhood trauma was associated with increased severity of positive, excitement/hostility, and depression/anxiety symptom dimensions. Importantly, BDNF variants significantly moderated these associations. Rs6265 (CT/TT genotypes) interacted with physical neglect to predict lower depression/anxiety scores, whereas rs11030101 (TA genotype) interacted with sexual abuse to predict increased depression/anxiety and showed negative interactions with physical neglect and total CTQ scores in relation to negative symptoms.

**Conclusion:**

These findings demonstrate that *BDNF* polymorphisms act as genetic modifiers of trauma-related symptom expression, supporting a gene–environment interaction model underlying phenotypic heterogeneity in schizophrenia.

## Introduction

Schizophrenia (SZ) is a highly heritable and phenotypically heterogeneous neuropsychiatric disorder (1–3). Genome-wide association studies have identified numerous risk loci; however, these genetic factors alone explain only a portion of disease liability (4). Increasing evidence indicates that genetic susceptibility interacts with environmental exposures to shape clinical phenotypes, consistent with a gene–environment interaction framework (5, 6).

Among environmental factors, childhood trauma represents one of the most robust non-genetic risk exposures for SZ (7–9). Early-life adversity has been associated not only with increased disease risk but also with marked variability in symptom expression, suggesting that trauma acts as a modifier of disease phenotype rather than a deterministic cause (10). However, individuals exposed to similar traumatic experiences often display divergent clinical outcomes, implying the presence of genetic moderators (11).

Brain-derived neurotrophic factor (BDNF) is a compelling candidate gene for mediating such effects (12–15). BDNF plays a central role in neuronal survival, synaptic plasticity, and stress-related neurodevelopment (16, 17). Functional polymorphisms within the *BDNF* gene, including rs6265 (Val66Met), rs2030324, and rs11030101, influence *BDNF* transcription, intracellular trafficking, and activity-dependent secretion. The rs2030324 polymorphism, alternatively designated as C270T, is situated within the promoter region of the *BDNF* gene and has been demonstrated to modulate transcriptional activity, thereby influencing BDNF expression levels. The rs11030101 variant, located in the intronic region of the *BDNF* gene, plays a crucial regulatory role in gene expression through potential effects on mRNA splicing and stability (18–20). Notably, the rs6265 polymorphism, commonly referred to as Val66Met (dbSNP: rs6265), results from a guanine to adenine substitution at nucleotide position 196, leading to an amino acid alteration from valine to methionine at codon 66 (Val66Met) in the BDNF protein. This non-synonymous single nucleotide polymorphism has been shown to significantly impact BDNF protein folding, intracellular trafficking, and activity-dependent secretion (21). These variants have been implicated in stress reactivity and psychiatric phenotypes, positioning *BDNF* as a potential genetic modifier of environmental adversity (22). Previous studies have suggested interactions between *BDNF* variants and childhood trauma in relation to emotional and cognitive outcomes; however, findings remain inconsistent, and data from first-episode schizophrenia are limited. Studying individuals early in the disease course offers a unique opportunity to reduce confounding effects of chronic illness and long-term medication exposure.

Therefore, the present study aimed to investigate whether common BDNF polymorphisms are associated with the relationship between childhood trauma and symptom dimensions in first-episode schizophrenia (FES), thereby clarifying the genetic architecture underlying trauma-related phenotypic heterogeneity.

## Materials and methods

### Subjects

This study was approved by the Ethics Committee of the Third People’s Hospital of Zhongshan, and written informed consent was obtained from all participants or their legal guardians. Patients with SZ were recruited between 2018 and 2022 and met DSM-5 diagnostic criteria for schizophrenia. Inclusion criteria were: (1) first episode of psychosis or medication-free for at least three months; (2) illness duration ≤2 years; (3) age between 16 and 60 years; and (4) absence of psychotic symptoms due to substance use, medical conditions, or other psychiatric disorders. Healthy controls were recruited from the same geographic region and had no personal or family history of psychiatric disorders. All controls were of Han ethnicity and underwent standardized physical and mental health assessments.

### Clinical and trauma assessments

Clinical symptoms were assessed using the Positive and Negative Syndrome Scale (PANSS) (23) by two trained psychiatrists with high inter-rater reliability (*r* > 0.80). Childhood trauma exposure was evaluated using the Childhood Trauma Questionnaire–Short Form (CTQ-SF) (24), which assesses five trauma subtypes: emotional neglect (EN), physical abuse (PA), emotional abuse (EA), sexual abuse (SA), and physical neglect (PN).

### Genotyping

Peripheral blood samples were collected for genomic DNA extraction. Three *BDNF* polymorphisms (rs6265, rs2030324, and rs11030101) were genotyped using the MassARRAY platform (Agena Bioscience). All loci were tested for Hardy–Weinberg equilibrium.

### Statistical analysis

Statistical analyses were performed using SPSS version 20.0. Group differences were examined using analysis of covariance (ANCOVA) or logistic regression, adjusting for age, sex, and education. Pearson correlation analyses were conducted to assess associations between CTQ scores and PANSS dimensions. Generalized linear models were used to examine gene–environment interaction effects, controlling for sex, age, education, age at onset, and family history. Statistical significance was set at *P* < 0.05.

## Results

### Demographic characteristics and candidate SNP information

A total 93 patients with FES and 64 healthy controls were included in the analysis. Sex distribution did not differ significantly between the two groups (*P* > 0.05; **Table 1**). Patients with SZ were significantly younger and had fewer years of education than healthy controls (both *P* < 0.05). The mean age at onset of SZ group was 23.78 ± 7.60 years.

**Table 1:** Comparison of demographic characteristics between the cases and controls.

Variables	Total (n = 157)	Control group (n = 64)	Study Group (n = 93)	Statistic	*P*
Age, Mean ± SD	40.86 ± 9.80	44.52 ± 8.41	38.34 ± 9.94	*t* = 4.06	**<.001**
Years of education, Mean ± SD	9.48 ± 3.46	9.92 ± 4.62	9.17 ± 2.33	*t* = 1.20	0.234
Sex, n(%)				*χ²* = 3.26	0.071
Male	106 (67.52)	38 (59.38)	68 (73.12)		
Female	51 (32.48)	26 (40.62)	25 (26.88)		

Bold represents *P* < 0.05.

Genomic DNA extracted from peripheral blood samples met quality requirements for genotyping. All three *BDNF* polymorphisms (rs6265, rs2030324, and rs11030101) conformed to Hardy–Weinberg equilibrium (*P* > 0.05), indicating reliable genotype distribution. Logistic regression analyses adjusted for age, sex, and years of education revealed no significant differences in genotype frequencies between patients and controls for any of the three loci (**Table 2**), suggesting the absence of main genetic effects on SZ diagnosis.

**Table 2:** Distribution of *BDNF* genotypes in schizophrenia cases and controls.

Gene locus	Genotype	*β*	SE	*X^2^*	*P*	OR(95%CI)
rs6265	CC					
	CT	-0.26	0.467	0.309	0.578	0.771(0.309~1.927)
	TT	0.356	0.544	0.427	0.514	1.427(0.491~4.149)
rs2030324	AA					
	GA	-0.026	0.394	0.005	0.946	0.974(0.45~2.108)
	GG	-0.219	0.573	0.146	0.702	0.803(0.262~2.467)
rs11030101	AA					
	TA	-0.326	0.377	0.747	0.387	0.722(0.345~1.511)
	TT	-0.783	1.179	0.441	0.507	0.457(0.045~4.609)

Adjusted for gender, age, years of education.

**Figure 1:**
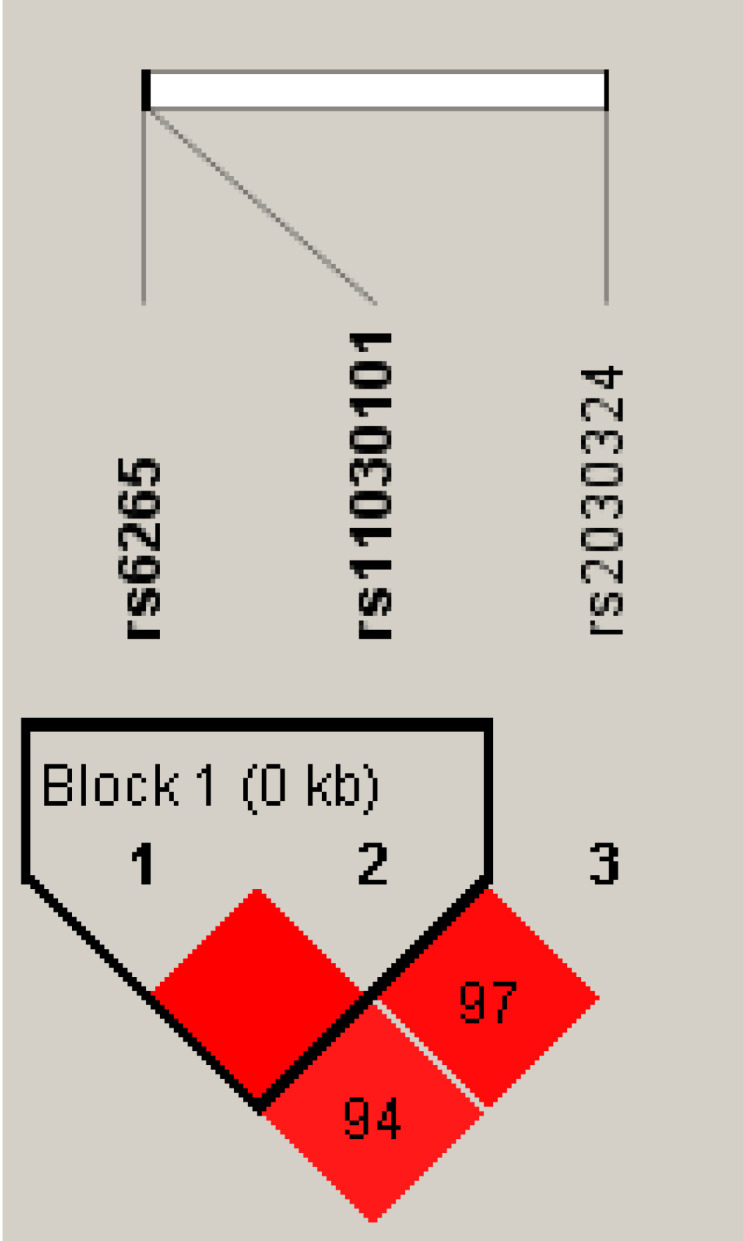
Linkage disequilibrium of BDNF polymorphisms. Pairwise linkage disequilibrium (D′) among rs6265, rs11030101, and rs2030324 in the study population. Red shading indicates stronger LD. Rs6265 and rs11030101 form a haplotype block (~1 kb), whereas rs2030324 lies outside the block.

Linkage disequilibrium analysis demonstrated strong non-random associations among the three BDNF polymorphisms, with D’ values ≥ 0.8 (**Figure 1**), supporting their combined evaluation in gene-environment interaction analyses.

### Childhood trauma exposure as an environmental risk factor

After adjustment for age, sex, and years of education, patients with SZ exhibited significantly higher scores across all Childhood Trauma Questionnaire (CTQ) subscales—including emotional neglect (EN), physical abuse (PA), emotional abuse (EA), sexual abuse (SA), and physical neglect (PN)—as well as higher total CTQ scores compared with healthy controls (all *P* < 0.05; **Table 3**). These findings confirm childhood trauma as a prominent environmental exposure in FES.

**Table 3:** Comparison of childhood trauma exposure (CTQ scores) between patients with first-episode schizophrenia and healthy controls.

Dimention	Group	Mean	S	*F*	*P*
EN	Study Group	11.89	4.57	22.135	<0.001
	Control group	8.41	3.58		
PA	Study Group	7.12	3.30	6.838	0.010
	Control group	5.91	1.26		
EA	Study Group	8.09	3.62	10.358	0.002
	Control group	6.25	2.05		
SA	Study Group	6.41	2.71	17.354	<0.001
	Control group	5.00	0.00		
PN	Study Group	10.86	3.97	12.931	<0.001
	Control group	8.55	3.11		
CTQ	Study Group	52.57	13.94	23.934	<0.001
	Control group	43.34	6.09		

Adjusted for gender, age, years of education; EN, emotional neglect; PA, physical abuse; EA, emotional abuse; SA, sexual abuse; PN, physical neglect.

When PANSS symptom dimensions were analyzed as quantitative psychiatric traits, childhood trauma exposure showed significant associations with symptom severity (**Table 4**). EN, PA, PN, and total CTQ scores were positively correlated with positive symptom severity (*r* = 0.211–0.286, all *P* < 0.05). EA was significantly associated with excitement/hostility (*r* = 0.221, *P* < 0.05). In addition, EN, EA, SA, PN, and total CTQ scores were significantly correlated with depression/anxiety severity (*r* = 0.213–0.384, all *P* < 0.05).

**Table 4:** Associations between childhood trauma (CTQ scores) and psychiatric symptom dimensions.

PANSS Factor		EN	PA	EA	SA	PN	CTQ
Negative symptoms	*r*	0.107	-0.051	-0.043	0.038	0.113	0.052
	*p*	0.309	0.625	0.682	0.718	0.282	0.620
Positive symptoms	*r*	0.250^*^	0.211^*^	0.103	0.150	0.258^*^	0.286^**^
	*p*	**0.015**	**0.043**	0.326	0.152	**0.013**	**0.005**
excitement/hostility	*r*	0.060	0.121	0.039	0.098	0.221^*^	0.119
	*p*	0.569	0.246	0.710	0.352	**0.034**	0.256
Anxiety/depression	*r*	0.213^*^	0.117	0.214^*^	0.222^*^	0.384^**^	0.313^**^
	*p*	**0.040**	0.266	**0.039**	**0.032**	**<0.001**	**0.002**
Cognitive impairment	*r*	0.136	0.061	-0.105	0.088	0.087	0.042
	*p*	0.193	0.563	0.314	0.403	0.408	0.688

EN, emotional neglect; PA, physical abuse; EA, emotional abuse; SA, sexual abuse; PN, physical neglect; *P < 0.05, **P < 0.01.

Bold represents *P* < 0.05.

No significant correlations were observed between childhood trauma measures and negative symptom or cognitive impairment dimensions, indicating domain-specific effects of childhood trauma on psychiatric phenotypes.

### Gene–environment interactions between BDNF variants and childhood trauma

**Figure 2:**
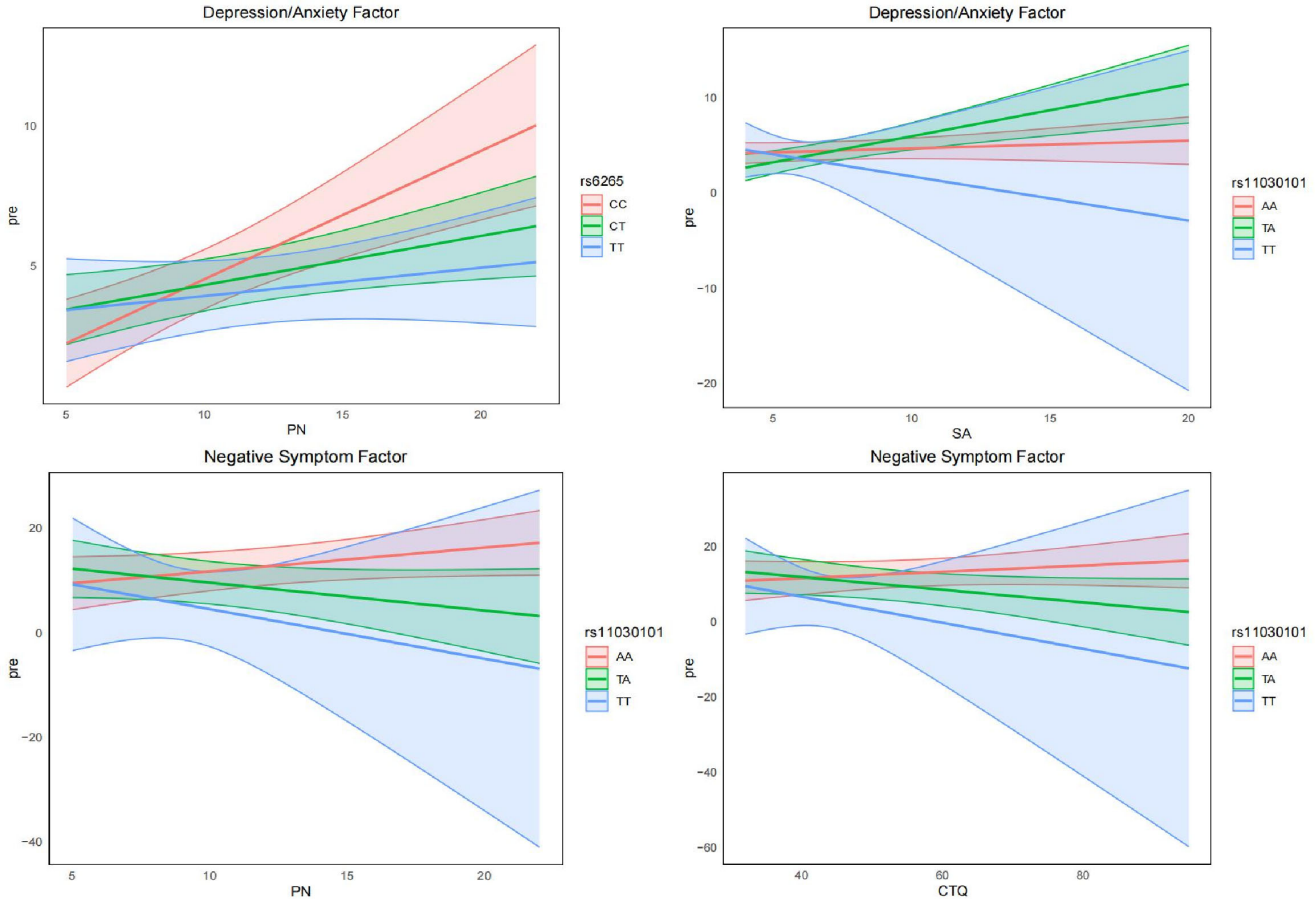
Gene–environment interactions between BDNF variants and childhood trauma on symptom dimensions. Interaction effects of rs6265 and rs11030101 with physical neglect (PN), sexual abuse (SA), and total CTQ scores on depression/anxiety and negative symptom factors. Lines represent predicted values by genotype, with shaded areas indicating 95% confidence intervals.

Generalized linear models were used to examine interactions between BDNF polymorphisms and childhood trauma exposure on psychiatric symptom dimensions, adjusting for sex, age, education, age at onset, and family history (**Tables 5**–**7**; **Figure 2**).

**Table 5:** Gene–environment interaction effects of *BDNF* polymorphisms (rs6265, rs2030324, and rs11030101) and childhood trauma (CTQ dimensions) on depression/anxiety symptoms.

Anxiety/depression								
rs6265			rs2030324			rs11030101		
Genotype	*β*	*P*		*β*	*P*		*β*	*P*
CC			AA			AA		
CT×EN	0.004	0.970	GA×EN	0.144	0.213	TA×EN	-0.009	0.933
TT×EN	-0.122	0.379	GG×EN	0.112	0.443	TT×EN	0.055	0.843
CC			AA			AA		
CT×PA	0.122	0.451	GA×PA	-0.142	0.463	TA×PA	0.080	0.559
TT×PA	0.173	0.521	GG×PA	-0.044	0.872	TT×PA	NA	NA
CC			AA			AA		
CT×EA	0.231	0.136	GA×EA	0.015	0.907	TA×EA	0.088	0.486
TT×EA	0.078	0.635	GG×EA	0.037	0.852	TT×EA	-0.022	0.963
CC			AA			AA		
CT×SA	0.139	0.512	GA×SA	-0.251	0.334	TA×SA	0.465	**0.010**
TT×SA	0.390	0.242	GG×SA	-0.274	0.400	TT×SA	-0.546	0.397
CC			AA			AA		
CT×PN	-0.283	**0.040**	GA×PN	0.153	0.186	TA×PN	-0.045	0.698
TT×PN	-0.357	**0.022**	GG×PN	0.007	0.970	TT×PN	-0.040	0.910
CC			AA			AA		
CT×CTQ	-0.020	0.600	GA×CTQ	0.012	0.746	TA×CTQ	-0.006	0.866
TT×CTQ	-0.035	0.452	GG×CTQ	0.003	0.949	TT×CTQ	0.005	0.966

Adjusted for gender, age, years of education, family history, and age at first onset. Bold represents *P* < 0.05.

**Table 6:** Gene–environment interaction effects of BDNF polymorphisms (rs6265, rs2030324, and rs11030101) and childhood trauma (CTQ dimensions) on positive symptom severity.

Positive symptoms								
rs6265			rs2030324			rs11030101		
Genotype	*β*	*P*		*β*	*P*		*β*	*P*
CC			AA			AA		
CT×EN	0.024	0.929	GA×EN	0.159	0.556	TA×EN	-0.138	0.578
TT×EN	-0.035	0.914	GG×EN	0.226	0.512	TT×EN	0.163	0.806
CC			AA			AA		
CT×PA	-0.297	0.422	GA×PA	-0.669	0.141	TA×PA	-0.391	0.232
TT×PA	0.809	0.191	GG×PA	-0.310	0.629	TT×PA	NA	NA
CC			AA			AA		
CT×EA	-0.261	0.481	GA×EA	-0.347	0.272	TA×EA	-0.294	0.343
TT×EA	0.253	0.523	GG×EA	-0.427	0.367	TT×EA	-0.274	0.815
CC			AA			AA		
CT×SA	-0.186	0.717	GA×SA	-0.703	0.263	TA×SA	-0.343	0.451
TT×SA	0.104	0.898	GG×SA	-0.458	0.560	TT×SA	-0.703	0.672
CC			AA			AA		
CT×PN	0.477	0.169	GA×PN	0.116	0.689	TA×PN	-0.033	0.910
TT×PN	0.284	0.467	GG×PN	0.380	0.396	TT×PN	-0.273	0.762
CC			AA			AA		
CT×CTQ	-0.006	0.950	GA×CTQ	-0.081	0.378	TA×CTQ	-0.101	0.219
TT×CTQ	0.055	0.626	GG×CTQ	0.003	0.982	TT×CTQ	0.027	0.931

Adjusted for gender, age, years of education, family history, and age at first onset.

**Table 7:** Gene–environment interaction effects of BDNF polymorphisms (rs6265, rs2030324, and rs11030101) and childhood trauma (CTQ dimensions) on excitement/hostility symptoms.

Excitement/hostility								
rs6265			rs2030324			rs11030101		
Genotype	*β*	*P*		*β*	*P*		*β*	*P*
CC			AA			AA		
CT×EN	-0.122	0.339	GA×EN	0.098	0.452	TA×EN	-0.005	0.967
TT×EN	-0.158	0.310	GG×EN	0.234	0.160	TT×EN	0.294	0.344
CC			AA			AA		
CT×PA	0.052	0.769	GA×PA	-0.189	0.371	TA×PA	-0.256	0.085
TT×PA	0.194	0.510	GG×PA	0.168	0.574	TT×PA	NA	NA
CC			AA			AA		
CT×EA	0.005	0.979	GA×EA	0.016	0.914	TA×EA	-0.140	0.314
TT×EA	-0.036	0.848	GG×EA	0.185	0.406	TT×EA	0.790	0.135
CC			AA			AA		
CT×SA	-0.191	0.417	GA×SA	-0.455	0.117	TA×SA	-0.031	0.881
TT×SA	0.199	0.590	GG×SA	-0.201	0.577	TT×SA	-0.010	0.990
CC			AA			AA		
CT×PN	-0.212	0.190	GA×PN	0.042	0.753	TA×PN	0.057	0.670
TT×PN	-0.241	0.187	GG×PN	0.175	0.402	TT×PN	0.306	0.455
CC			AA			AA		
CT×CTQ	-0.028	0.535	GA×CTQ	-0.018	0.683	TA×CTQ	-0.040	0.296
TT×CTQ	-0.014	0.797	GG×CTQ	0.055	0.372	TT×CTQ	0.134	0.352

Adjusted for gender, age, years of education, family history and age at first onset.

Significant gene–environment interactions were identified for rs6265 and rs11030101. Specifically, rs6265 CT and TT genotypes interacted with physical neglect to predict lower depression/anxiety scores (both *P* < 0.05), indicating a genotype-dependent attenuation of affective symptom expression. In contrast, the rs11030101 TA genotype interacted with sexual abuse to predict increased depression/anxiety severity (*P* = 0.010). Moreover, rs11030101 TA genotype showed significant negative interactions with physical neglect and total CTQ scores in relation to negative symptom severity, suggesting modulation of this psychiatric trait by cumulative trauma exposure. No significant interaction effects were observed for rs2030324 across symptom dimensions.

## Discussion

In this study, we investigated whether common *BDNF* polymorphisms influence the relationship between childhood trauma and psychiatric symptom dimensions in FES. While childhood trauma exerted significant main effects on affective and positive symptom dimensions, our findings demonstrate that *BDNF* genetic variation critically shapes the magnitude and direction of these associations, supporting a gene–environment interaction model underlying phenotypic heterogeneity.

Consistent with prior evidence (25, 26), childhood trauma was strongly associated with depression/anxiety and positive symptom severity. These associations were domain-specific, with minimal effects on negative symptoms or cognitive impairment when considered independently. This pattern suggests that early-life adversity preferentially impacts stress-sensitive affective and psychotic dimensions, rather than core deficit domains, at least during the early stage of illness (27).

Importantly, we observed significant moderating effects of *BDNF* polymorphisms. The rs6265 (Val66Met) variant interacted with physical neglect to predict reduced depression/anxiety severity among Met allele carriers (28). Although the Met allele has often been associated with increased stress vulnerability, accumulating evidence indicates that its functional consequences are context-dependent. Under certain environmental conditions, altered activity-dependent BDNF secretion may engage compensatory neuroplastic mechanisms, resulting in attenuated affective symptom expression (28). Our findings support a modifier model in which rs6265 does not confer direct disease risk but influences phenotypic sensitivity to environmental stressors.

In contrast, rs11030101 emerged as a distinct genetic moderator (19, 29). The TA genotype interacted with sexual abuse to exacerbate depression/anxiety symptoms and showed inverse interactions with physical neglect and cumulative trauma exposure in relation to negative symptom severity. Given its location within a regulatory region of the *BDNF* gene, rs11030101 may influence transcriptional efficiency or mRNA processing, thereby altering stress-responsive BDNF signaling. These results suggest that different *BDNF* variants may modulate discrete psychiatric phenotypes through partially overlapping but functionally distinct mechanisms.

From a psychiatric genetic perspective, these gene–environment interactions likely converge on stress-sensitive neurodevelopmental pathways (30). We hypothesize that childhood trauma may activate the hypothalamic–pituitary–adrenal axis, potentially leading to altered glucocorticoid dynamics which could influence BDNF-related signaling and fronto-limbic circuit function (31, 32). Genetic variation within *BDNF* might modify such processes by affecting neurotrophic availability or signaling efficiency, thereby contributing to individual differences in symptom presentation (33). However, this proposed pathway remains speculative; future studies incorporating direct measures of peripheral BDNF, cortisol levels, and neuroimaging markers are needed to empirically test these mechanisms.

Several limitations should be acknowledged. Childhood trauma was assessed retrospectively and may be subject to recall bias. Given the exploratory nature of this study and the modest sample size, correction for multiple testing was not applied, and the findings should be interpreted cautiously. Additionally, all participants were of Han Chinese ancestry, which minimizes population stratification but may limit generalizability. Future studies with larger, ethnically diverse cohorts and longitudinal designs are warranted to validate these findings and clarify the molecular mechanisms underlying BDNF-mediated genetic modulation.

In summary, our results indicate that *BDNF* polymorphisms function as genetic modifiers of trauma-related psychiatric phenotypes rather than primary risk loci. These findings reinforce the importance of gene–environment interactions in shaping symptom heterogeneity in schizophrenia and provide a genetic framework for understanding individual differences in vulnerability to early-life adversity.

## Data Availability

The raw data supporting the conclusions of this article will be made available by the authors, without undue reservation.
